# Supplementary figures and images for: Effect of post-implant exercise on tumour growth rate, perfusion and hypoxia in mice
Source: PLoS One. 2020 Mar 18;15(3):e0229290. doi: 10.1371/journal.pone.0229290 (PMC7080225; doi:10.1371/journal.pone.0229290)

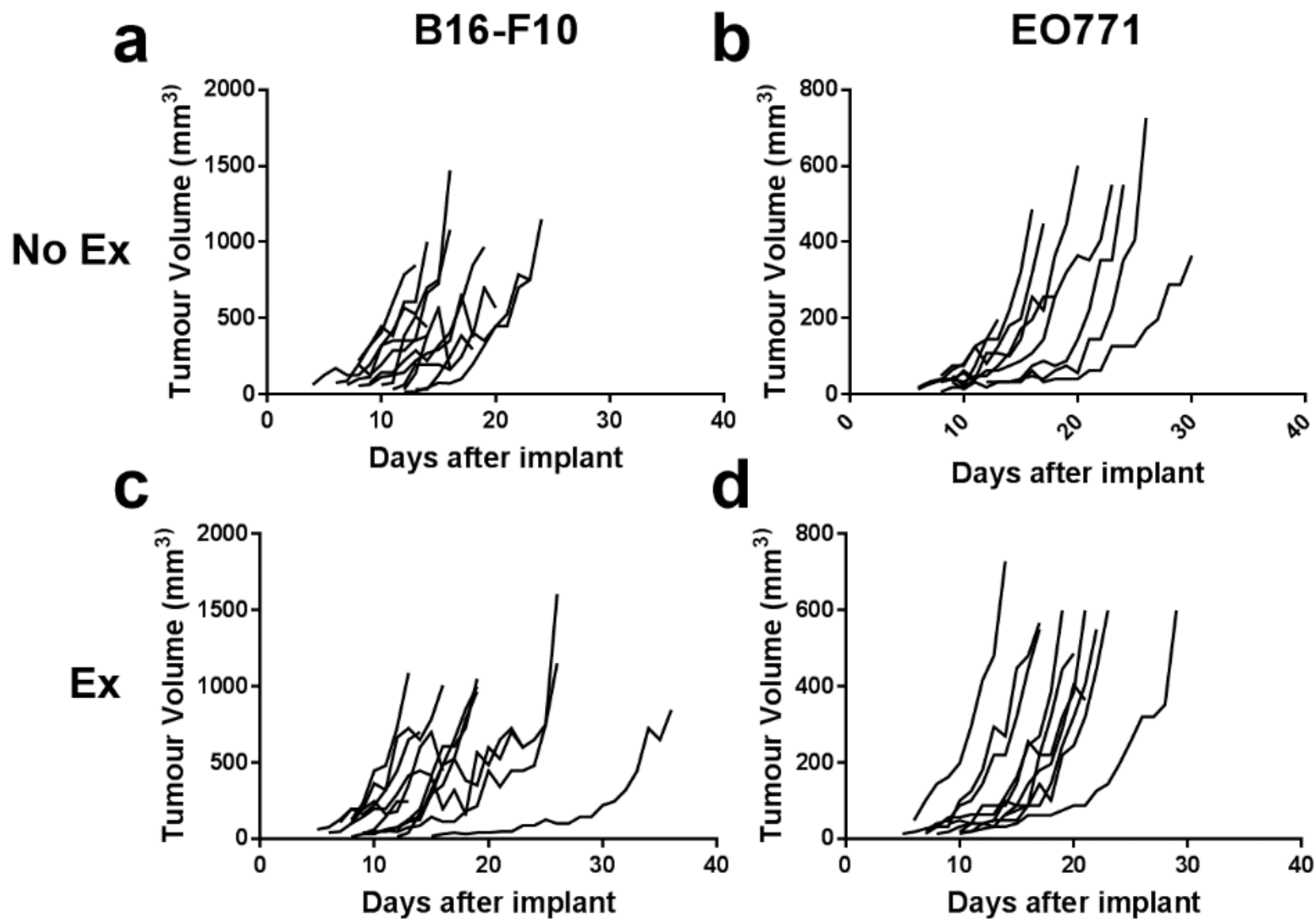

Supplement: S1 Fig — B16-F10: n-12 per group. EO771: n = 10 per group. (PDF) [file pone.0229290.s001.pdf]

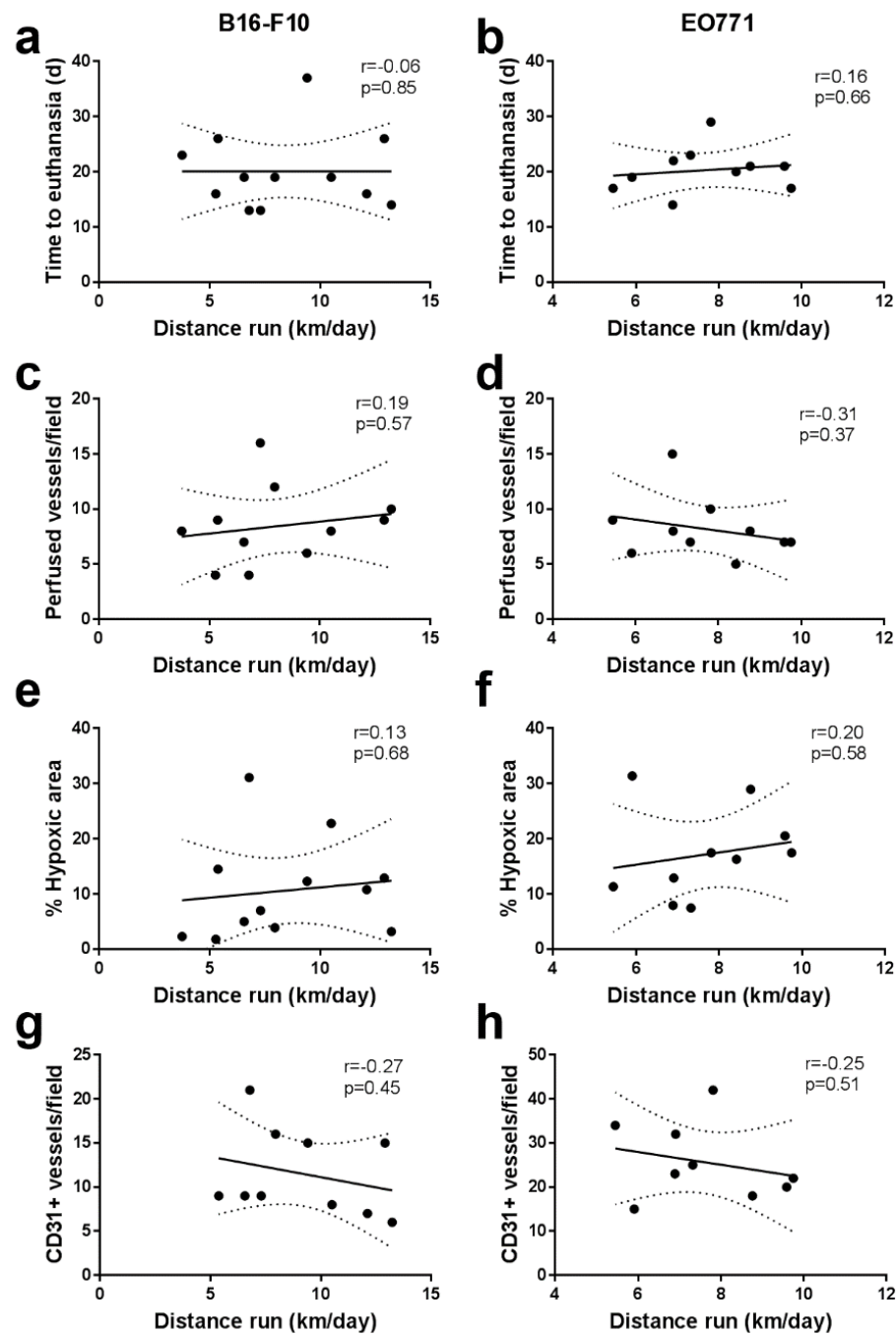

Supplement: S2 Fig — Correlation of the time to euthanasia (due to maximum tumour size) with average daily running distance in mice with B16-F10 (a) or EO771 (b) tumours. Correlation of perfused vessel number with average daily running distance in mice with B16-F10 (c) or EO771 (d) tumours. Correlation of hypoxic area with average daily running distance in mice with B16-F10 (e) or EO771 (f) tumours. Correlation of CD31+ vessel number with average daily running distance in mice with B16-F10 (g) or EO771 (h) tumours. Data analysed by Pearson (b, c, f, g, h) or Spearman correlation (a, d, e). Data shown as scatter plot with best fit line with 95% CI bands. B16-F10: n = 10–12, EO771: n = 9–10. (PDF) [file pone.0229290.s002.pdf]

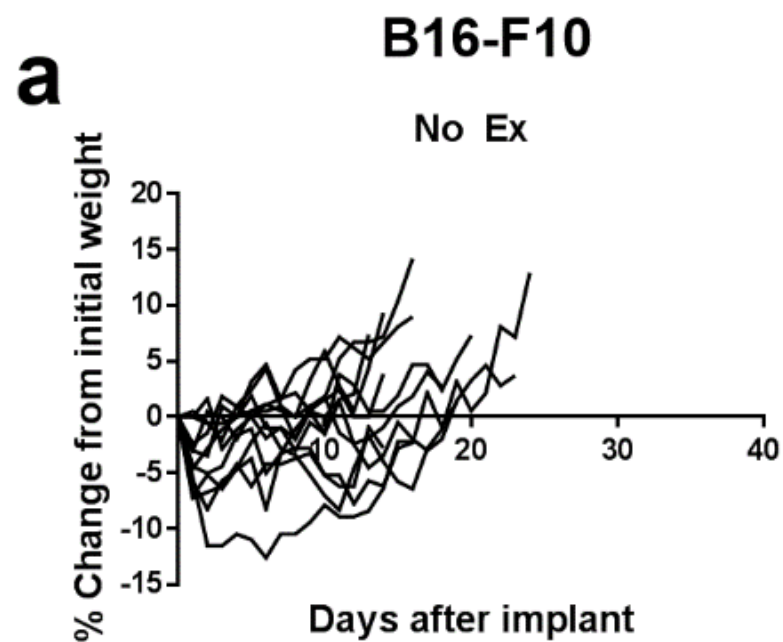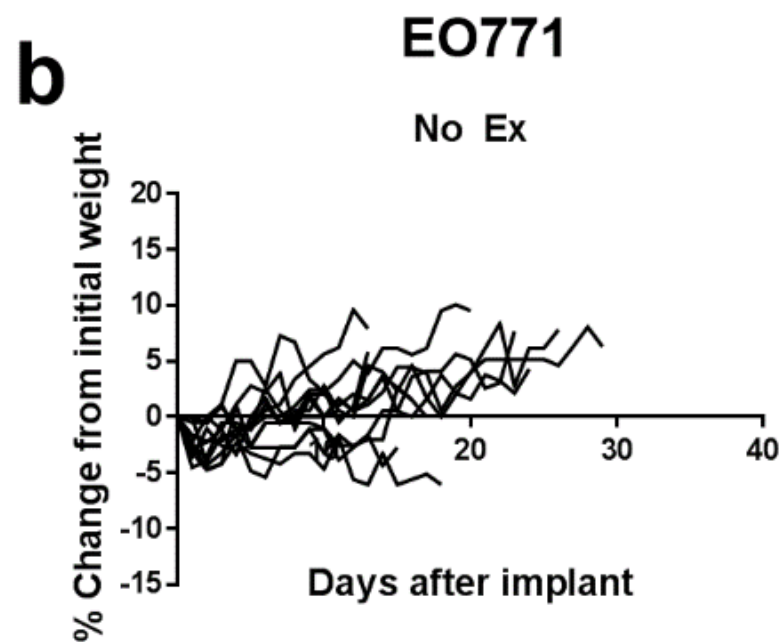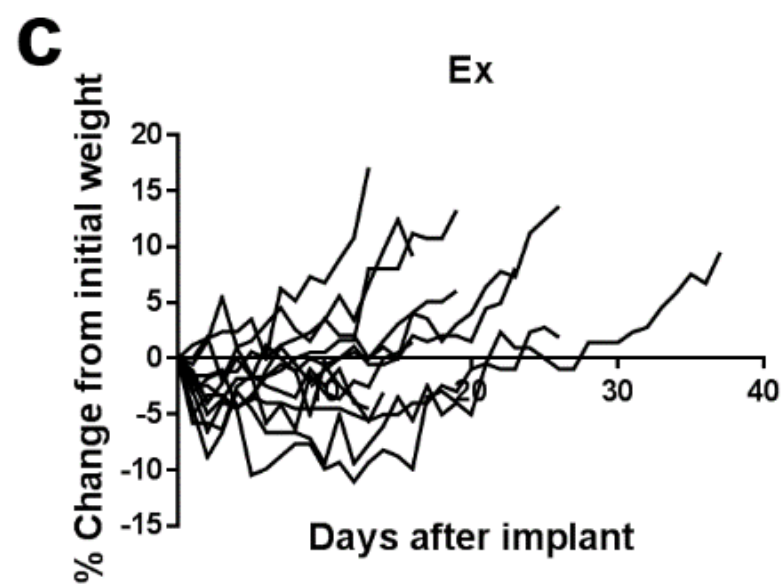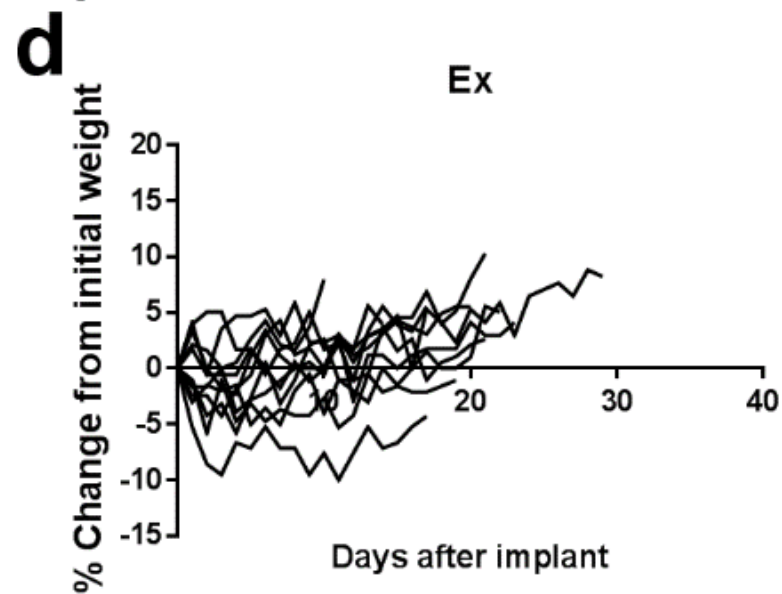

Supplement: S3 Fig — Individual body weight change over time for non-exercising (a, b) and exercising (c, d) mice bearing B16-F10 (a, c) or EO771 tumours (b, d). Weight change percentage uncorrected for tumour weight. B16-F10 body weight change: n = 12 per group; EO771 body weight change: n = 11–12. (PDF) [file pone.0229290.s003.pdf]

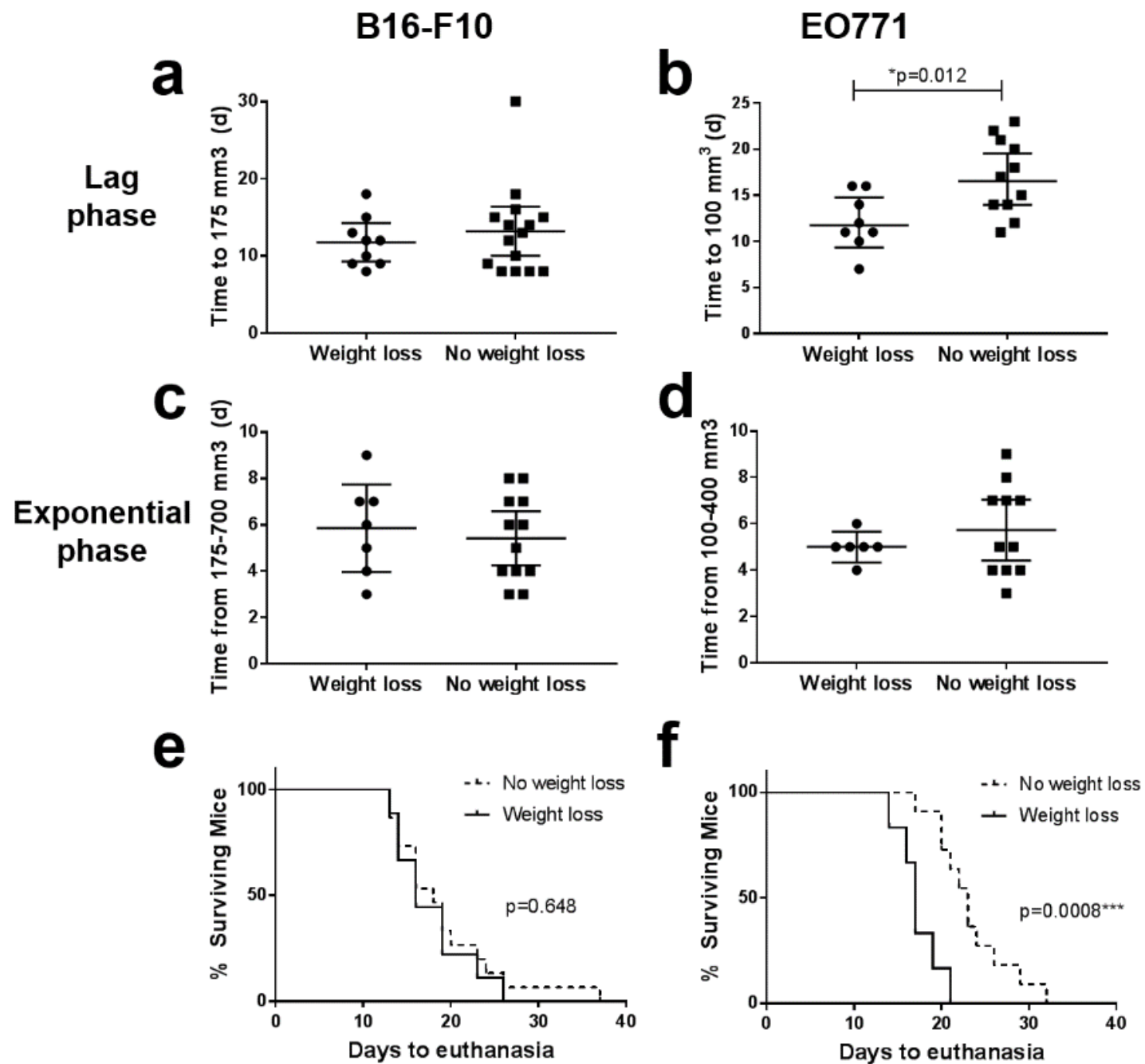

Supplement: S4 Fig — Tumour establishment time (lag phase, time to 175 or 100 mm3) in mice with B16-F10 (a) or EO771 (b) tumours according to mouse weight change. Exponential tumour growth rate (time for the tumour to quadruple in volume) in mice that did or did not lose weight with B16-F10 (c) or EO771 (d) tumours. Data are shown as individual data points and mean ± 95% CI. Data analysed using a two-tailed students t test. B16-F10 lag phase weight loss: n = 9, no weight loss: n = 15; EO771 lag phase weight loss: n = 8, no weight loss: n = 11; B16-F10 exponential phase weight loss: n = 7, no weight loss: n = 12; EO771 exponential phase weight loss: n = 6, no weight loss: n = 11. Survival curves for mice with or without weight loss while bearing B16-F10 (e) or EO771 (f) tumours. Animals were included in survival analysis only if euthanasia was due to tumour burden. Data analysed using Log-rank test. (PDF) [file pone.0229290.s004.pdf]

**B16-F10**

**EO771**

**20x**

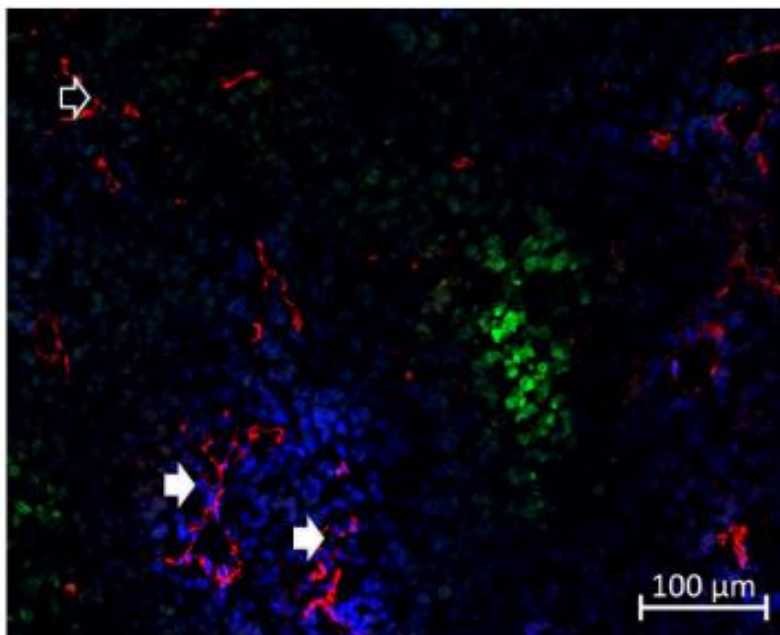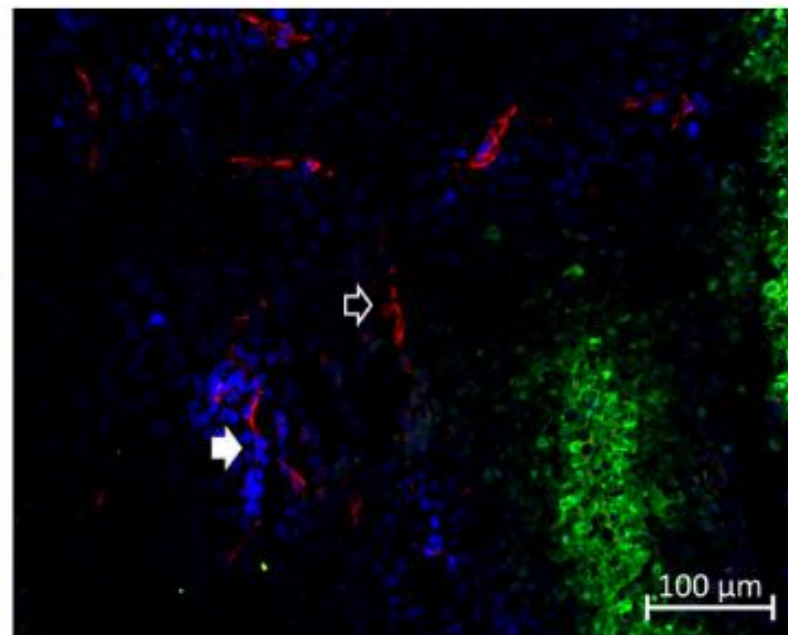

**40x**

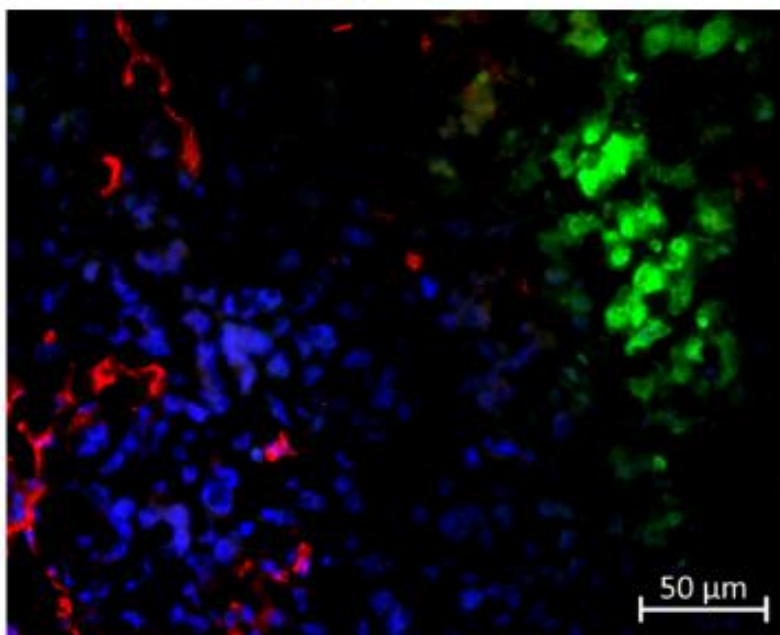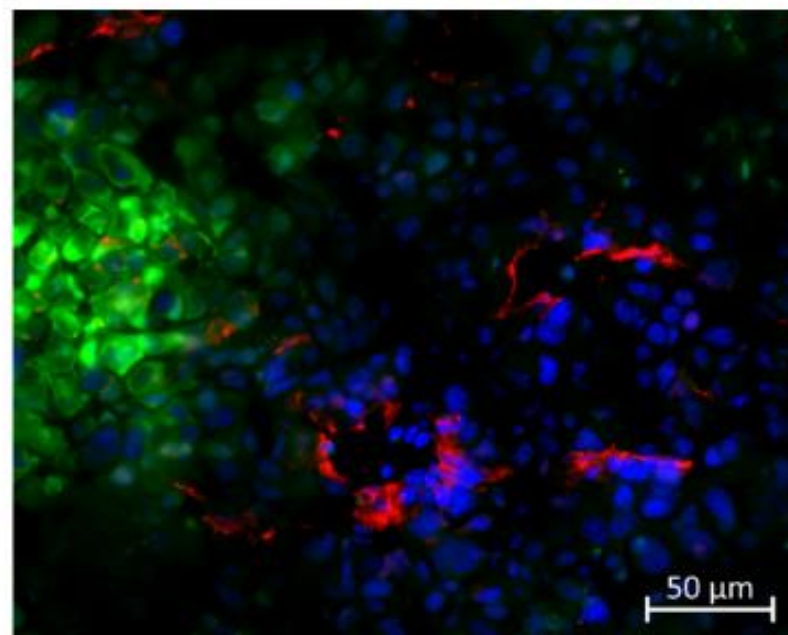

Supplement: S5 Fig — Red: CD31, green: pimonidazole, blue: Hoechst 33342. Closed arrows indicate examples of perfused CD31+ vessels and open arrows indicate examples of unperfused CD31+ vessels. Images are at 20x or 40x magnification as indicated. (PDF) [file pone.0229290.s005.pdf]

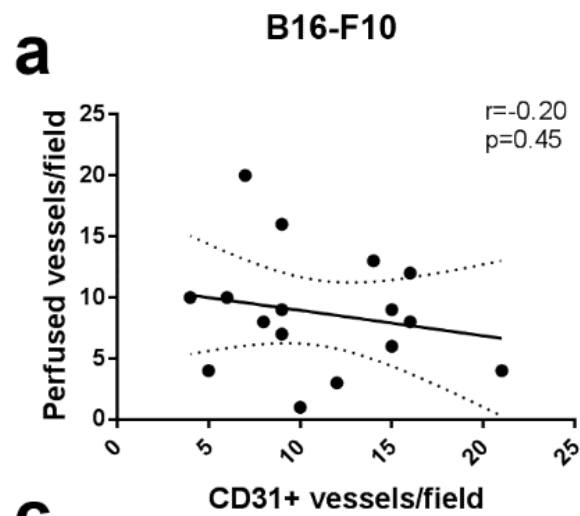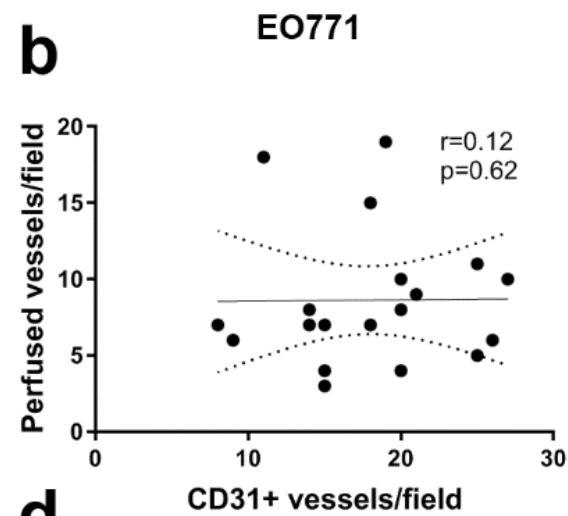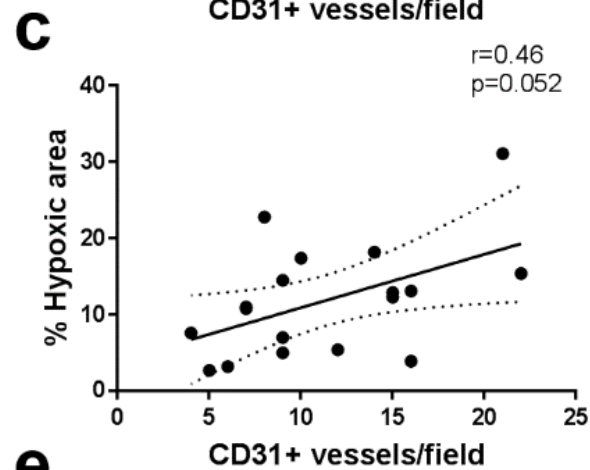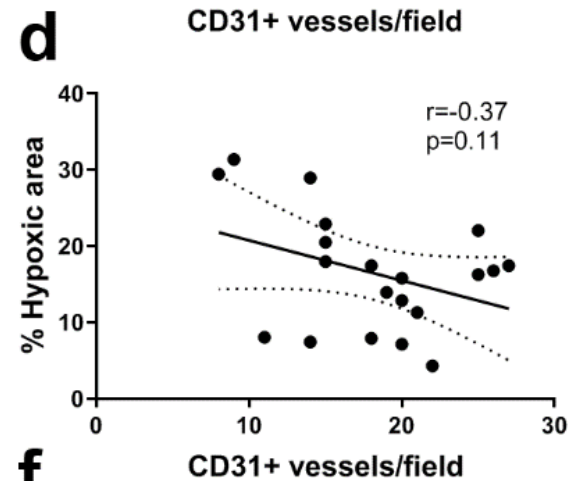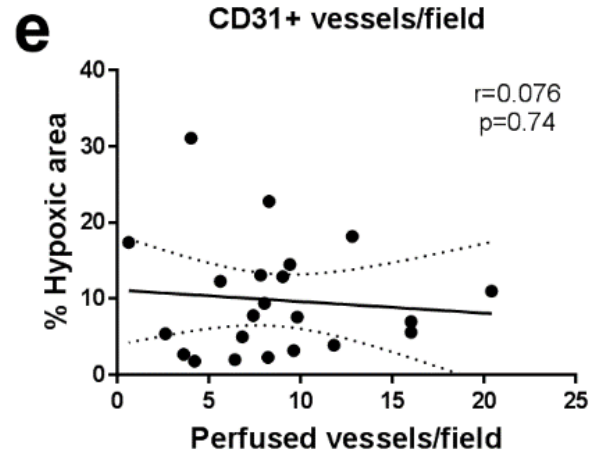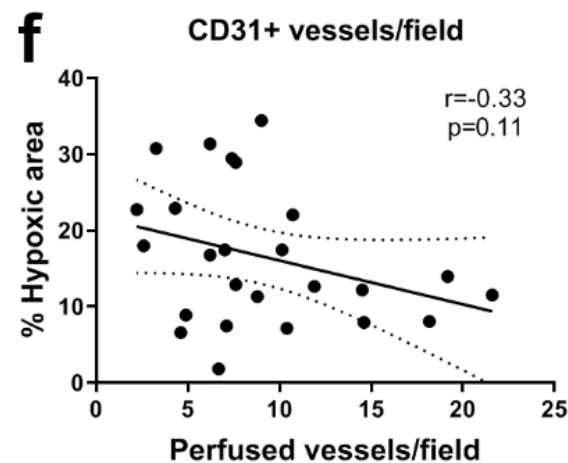

Supplement: S6 Fig — Correlation of perfused vessel number with CD31+ vessel number in B16-F10 (a) or EO771 (b) tumours. Correlation of hypoxia with CD31+ vessel number in B16-F10 (c) or EO771 (d) tumours. Correlation of hypoxic area with perfused vessel number in B16-F10 (e) or EO771 (f) tumours. Data analysed by Pearson (a, b, d, f) or Spearman correlation (c, e). Data shown as scatter plot with best fit line with 95% CI bands. B16-F10: perfused vs CD31 vessels n = 16; hypoxia vs CD31 vessels n = 18; hypoxia vs perfusion n = 22; EO771: perfused vs CD31 vessels n = 25; hypoxia vs CD31 vessels n = 18; hypoxia vs perfusion n = 19. (PDF) [file pone.0229290.s006.pdf]
