# Supplementary material for: Effect of post-implant exercise on tumour growth rate, perfusion and hypoxia in mice
Source: PLoS One. 2020 Mar 18;15(3):e0229290. doi: 10.1371/journal.pone.0229290 (PMC7080225; doi:10.1371/journal.pone.0229290)
Supplement: Attachment — Submitted filename: Response to Reviewers.docx [file pone.0229290.s008.docx]

Below are our detailed responses to the journal requirements and reviewers’ comments (indented in blue).

**Journal Requirements:**

1. When submitting your revision, we need you to address these additional requirements.
Please ensure that your manuscript meets PLOS ONE's style requirements, including those for file naming.

We have amended style as requested, although we did not find many issues and are happy to change this further if you provide us with details.

2. Please include a caption for figure 5.

Amended (line 296).

3. Thank you for stating the following in the Financial Disclosure section:

This research was funded in part by the Mackenzie Charitable Foundation, NZ. We would also like to acknowledge Dr. Andreas Moeller (QIMR Berghofer, Australia) for the kind gift of the EO771 cells and Andrew Dachs (Decision Consulting Ltd, NZ) for developing the magnetic counter for the mouse wheels. The funders had no role in study design, data collection and analysis, decision to publish, or preparation of the manuscript.

We note that you received funding from a commercial source: Decision Consulting Ltd, NZ

Please provide an amended Competing Interests Statement that explicitly states this commercial funder, along with any other [relevant declarations](https://apc01.safelinks.protection.outlook.com/?url=http%3A%2F%2Fwww.plosone.org%2Fstatic%2Fcompeting.action&data=02%7C01%7Cgabi.dachs%40otago.ac.nz%7C74befcf0da0142ffd5ae08d77ceb1d13%7C0225efc578fe4928b1579ef24809e9ba%7C1%7C1%7C637115221597537114&sdata=qjd8MeviD3ZRiczFobw%2Fmn%2BxCFBvZ8Oj%2FWVL9YsSwYM%3D&reserved=0) relating to employment, consultancy, patents, products in development, marketed products, etc.

This has been a misunderstanding. We did not receive any funding from a commercial source (Decision Consulting provided the modified mouse wheels), and have amended the manuscript accordingly (added line 125, removed lines 420-421). We therefore do not need to amend the Competing interest Statement.

**Reviewer #1**: This is a nice study evaluating the effect of exercise (running wheel) on tumor growth and vascular characteristics using B16F10 and E0771 models. Strengths of the work include the use of orthotopic models, appropriate sample size and statistical analysis, and the use of experimental design that minimizes animal stress. Also, this work is important because it reports a non-significant impact of exercise on tumor growth/vascular biology, which is important in the current climate of exercise oncology research where negative results are often ignored. Overall, I would like to see the study published, but do have some concerns which need to be addressed.

1. Abstract Line 20 “This has only been shown in breast and prostate cancer models…” seems a little inaccurate. Several studies have shown increases in tumor vessel perfusion and/or hypoxia. Perhaps soften the wording to "has been shown most convincingly in breast and prostate cancer models..."

Amended as requested (line 18).

For interest, could we please be provided with references to the studies mentioned that show increases in perfusion and/or reduction in hypoxia with exercise in models other than breast or prostate cancer? The only studies we are aware of which use a different tumour type are those by Schadler *et al*. in 2016 and Bedoya *et al.* in 2019 which use pancreatic models.

2. Mice received an exercise wheel on the day of tumor transplant. It’s not clear to me what clinical situation this is meant to mimic. Typically, patients are either already exercisers who then develop cancer (modeled by pre-implantation training) or will begin exercise after a cancer diagnosis (modeled by exercise beginning after a detectable/measurable tumor is present). Exercise beginning on the day of tumor cell inoculation is not an obvious model for me. Please explain in your introduction or discussion, or if there is no explanation, please list as a study limitation in the discussion.

We have clarified our model, see lines 133-135. Tumours were typically detectable within 5-10 days of implant. Here, we aimed to mimic patients starting exercise after (an early) cancer diagnosis. Cancer development may take years, whereas implantation of tumour cells is akin to early detection of a small mass.

3. Were mice that were euthanized early included in the analysis of vasculature and hypoxia?

Yes, clarified in revised manuscript (lines 144-146).

4. Others have shown in B16F10 tumors that exercise did not change vessel density, but did change the average length of the vessels or the number of open lumens. Did the authors evaluate these parameters in either tumor model?

We considered undertaking these analyses. However, we feel that neither of these parameters can be reliably quantified using immunohistochemical methods, due to the fact that thin tissue sections capture cuts through vessels in varying orientations (lengthwise, cross-section etc.) and it is therefore extremely difficult to accurately determine factors such as vessel length and number of open lumens.

5. There appears to be significant variation in the distance run between individual mice. Is it possible to analyze tumor growth or vascular characteristics in a way that takes this variation into consideration? For example, perhaps tumors behave differently in low exercising vs high exercising mice. Is there any correlation between amount of daily running and survival time, hypoxia, or vessel number?

These are valid suggestions. We have performed the proposed analyses and added them as a new supplementary figure, but they did not show associations with running distance (line 192-193, line 277-279, new S2 Fig).

6. I am concerned about the conclusions that were drawn regarding vascular structure, function, etc. based on images that are very difficult to see and interpret. In figure 3a, the images are extremely blurry. It would be nice to see both a low magnification and high magnification image for each condition. Also, how do the authors know that the Hoecsht 3342 is demonstrating perfused tumor vessels and not leakage outside of vessels? These images would be strengthened greatly by the addition of CD31 or VE-cadherin immunoflourescent staining so that the location of Hoecsht 3342 and of hypoxyprobe-1 relative to endothelium can truly be assessed. (3 color staining showing Hoecsht, hypoxia, and endothelium together)

To confirm the validity of using Hoechst33342 as perfusion marker, we have added representative images at low and high magnification of the requested 3-colour stain with CD31 in red, pimonidazole in green and Hoechst 33342 in blue (line 258-260, new S5 Fig). In addition, Hoechst 33342 has previously been validated for use as a perfusion marker in tumours (Smith *et al*. 1988). We apologise for the blurry images in the pdf – this appears to be a technical problem when converting the TIFF images into pdf online. The images used for analysis were not blurry and were clearly analysable.

We have send a compilation pdf version of all figures and supplementary figures, showing clear images of all figures.

7. Similarly, it’s not clear to me how the hypoxic area was determined? What software was used to determine the % hypoxic area? Is this a ratio of pimonidazole positive: total nuclei or pimonidazole: total tissue area? It seems that comparing to the total nuclei would be most appropriate because some of the tissue area may be necrotic, but if this is not possible due to software limitations, please explain the process for determining the % hypoxic area.

Hypoxia was determined by area while necrotic areas were excluded, as described (lines 171-173).

8. The figure legend for Figure 5 is labeled as Figure 1.

This error has been corrected.

**Reviewer #2**: This paper describes the results of a study of post-implantation exercise in syngeneic mouse tumour graft using B16-F10 melanoma cells and EO771 breast cancer cells. No differences were observed including in measures of tumour growth, and hypoxic fraction and vascular density at sacrifice.

The work appears to have been conducted carefully, and should be published – not least in the interests of avoiding publication bias. The discussion is well balanced.

I have a few relatively minor points for clarification.

In figure 1, the y-axis is labelled as cumulative survival. This is potentially a little confusing as all animals were subjected to pre-specified euthanasia end-points (I think). In this case, it would be better to simply refer to % reaching end-point. Could the authors also clarify whether all end-points were reached due to tumour size, or where some were due to weight loss or other criteria?

All endpoints used in the survival analysis were euthanasia due to tumour size, and figure legend has been altered accordingly (line 199-200). Mice euthanised due to other endpoints are described in lines 142-147 and were excluded from the survival analysis.

In figure 2, the mean distance run does not appear to be reduced with tumour growth. Does this suggest a relative low systemic effect of the malignancy? Could the authors comment?

Due to the observation that we had relatively low numbers of cachectic mice, we agree that the systemic effect of our chosen malignancies and early endpoints are likely to be comparatively low. However, we have unpublished analyses on muscle tissue which indicate that there is some effect of these tumours on distal tissues, even though running behaviour is not affected. It is unclear how running behaviour is affected in mice with tumours that cause high rates of cachexia (which one could assume to have a high systemic effect), as cancer cachexia + exercise studies often use treadmill running or do not report voluntary wheel running distance. .

In figure 3, the hypoxic area is extremely variable. Could the authors describe this further? In any one tumour, were the 10 random fields similar? Or was the variability observed within tumours?

We have clarified the method used for analysis of hypoxia (lines 171-173). Hypoxia was more variable between tumours than within individual tumours (mean SD of hypoxic fraction of individual fields used for analysis: 4.96 and 7.8 for B16-F10 and EO771, respectively, vs SD of mean hypoxic fraction between tumours: 7.27 and 9.69 for B16-F10 and EO771, respectively).

In figures 3 and 4, as indicated in the legends, there are slightly different numbers. Could the authors indicate why?

There were a number of reasons as to why mice were not included in the histological analyses, reducing the final number used in analysis from the original 12. These were due to early euthanasia (line 142-147) or due to technical issues (line 163-165), as follows:

Fig 3:

EO771 No Ex: two mice had intra-peritoneal tumours and one tumour exhibited low-level, diffuse perfusion which could not be quantified accurately by vessel count, bringing the number of tumours used for analysis to n=9.

EO771 Ex: One mouse had intra-peritoneal tumours, and one was euthanised before tumour development due to pyometra, bringing the number of tumours used for analysis to n=10.

Fig 4:

The numbers for the CD31 stain are lower for the B16-F10 tumours as we encountered technical difficulty in measuring staining in some of the tumours. In a number of melanomas, dark pigmentation was observed, making true DAB staining difficult to identify and quantification unreliable. For this reason, these tumours were excluded from the CD31 analysis. Four tumours were removed from analysis in the B16-F10 No Ex group, and two from the B16-F10 Ex group for this reason, bringing the numbers to n=8 and n=10, respectively.

As mentioned above, two mice in the EO771 No Ex group had peritoneal tumours, bringing the number of useable tumours down to n=10. In the EO771 Ex group, one mouse had intra-peritoneal tumours and one was euthanised early due to pyometra, bringing the total down to n=10.
