# Supplementary material for: Effect of post-implant exercise on tumour growth rate, perfusion and hypoxia in mice
Source: PLoS One. 2020 Mar 18;15(3):e0229290. doi: 10.1371/journal.pone.0229290 (PMC7080225; doi:10.1371/journal.pone.0229290)
Supplement: Attachment — Submitted filename: Response to Reviewers V2.docx [file pone.0229290.s009.docx]

Below are our detailed responses to the reviewer’s comment (indented in blue).

Reviewer 2.

'However they do not appear to have adjusted the manuscript to make these issues clear in the revision. For instance, in the clarifications, I suggested, for the figure legends, it would seem reasonable to make the answers to the questions explicit with a simple sentence. I do not regard this as mandatory, but do feel the authors would improve the clarity of the manuscript if they did this. Otherwise the reader will puzzle over the issues which I raised in just the same way as myself.'

We have made the following changes in response to the reviewer’s comments:

Line 200	Fig 1 legend, added: ‘mice euthanised due to other endpoints were excluded from the survival analysis’

Line 211	added: ‘and that the systemic effects are comparatively low’

Line 264	added: ‘Hypoxia was more variable between tumours than within individual tumours (mean SD of hypoxic fraction of individual fields used for analysis: 4.96 and 7.8 for B16-F10 and EO771, respectively, vs SD of mean hypoxic fraction between tumours: 7.27 and 9.69 for B16-F10 and EO771, respectively).’

Line 278	Fig 3 legend, added: ‘B16-F10 No Ex and Ex: n=12, EO771 No Ex: n=9 (two mice had intra-peritoneal tumours and one tumour exhibited low-level, diffuse perfusion which could not be quantified) and Ex: n=10 (one mouse had intra-peritoneal tumours, and one was euthanised before tumour development due to pyometra).’

Line 292	Fig 4 legend, added: ’ B16-F10 No Ex: n=8 (four tumours could not be quantified due to dark pigmentation), B16-F10 Ex: n=10 (two tumours could not be quantified due to dark pigmentation), EO771 No Ex: n=10 (two mice had intra-peritoneal tumours) and Ex: n=10 (one mouse had intra-peritoneal tumours, and one pyometra).’

Line 311	Fig 5 legend, added: ‘EO771: n=10 per group (three mice had intra-peritoneal tumours, and one pyometra).’

Line 331	Fig 6 legend, added: ‘B16-F10: n=24, EO771: n=20 (three mice had intra-peritoneal tumours, and one pyometra). (b) Comparison of the number of CD31+ vessels in B16-F10 and EO771 tumours. B16-F10: n=18 (six tumours could not be quantified due to dark pigmentation), EO771: n=20 (three mice had intra-peritoneal tumours, and one pyometra). Quantification of hypoxia (c) and perfusion (d) in B16-F10 vs EO771 tumours. B16-F10: n=24, EO771: n=20 (three mice had intra-peritoneal tumours, and one pyometra).’
